# Understanding the Role of Values in Health Policy Decision-Making From the Perspective of Policy-Makers and Stakeholders: A Multiple-Case Embedded Study in Chile and Colombia

**DOI:** 10.15171/ijhpm.2019.94

**Published:** 2019-11-05

**Authors:** Marcela Vélez, Michael G. Wilson, Julia Abelson, John N. Lavis, Guillermo Paraje

**Affiliations:** ^1^McMaster Health Forum, McMaster University, Hamilton, ON, Canada.; ^2^Department of Health Research Methods, Evidence and Impact, McMaster University, Hamilton, ON, Canada.; ^3^Centre for Health Economics and Policy Analysis, McMaster University, Hamilton, ON, Canada.; ^4^Health Policy Ph.D. Program, McMaster University, Hamilton, ON, Canada.; ^5^Department of Paediatrics, Faculty of Medicine, University of Antioquia, Antioquia, Colombia.; ^6^Department of Political Science, McMaster University, Hamilton, ON, Canada.; ^7^Africa Centre for Evidence, University of Johannesburg, Johannesburg, South Africa.; ^8^Business School, Universidad Adolfo Ibáñez, Santiago, Chile.

**Keywords:** Chile, Colombia, Values, Health System Financing, Decision-Making

## Abstract

**Background:** Chile and Colombia are examples of Latin American countries with health systems shaped by similar values. Recently, both countries have crafted policies to regulate the participation of private for-profit insurance companies in their health systems, but through very different mechanisms. This study asks: what values are important in the decision-making processes that crafted these policies? And how and why are they used?

**Methods:** An embedded multiple-case study design was carried out for 2 specific decisions in each country: (1) in Chile, the development of the Universal Plan of Explicit Entitlements -AUGE/GES - and mandating universal coverage of treatments for high-cost diseases; and (2) in Colombia, the declaration of health as a fundamental right and a mechanism to explicitly exclude technologies that cannot be publicly funded. We interviewed key informants involved in one or more of the decisions and/or in the policy analysis and development process that contributed to the eventual decision. The data analysis involved a constant comparative approach and thematic analysis for each case study.

**Results:** From the 40 individuals who were invited, 28 key informants participated. A tension between 2 important values was identified for each decision (eg, solidarity vs. individualism for the AUGE/GES plan in Chile; human dignity vs. sustainability for the declaration of the right to health in Colombia). Policy-makers used values in the decisionmaking process to frame problems in meaningful ways, to guide policy development, as a pragmatic instrument to make decisions, and as a way to legitimize decisions. In Chile, values such as individualism and free choice were incorporated in decision-making because attaining private health insurance was seen as an indicator of improved personal economic status. In Colombia, human dignity was incorporated as the core value because the Constitutional Court asserted its importance in its use of judicial activism as a check on the power of the executive and legislative branches.

**Conclusion:** There is an opportunity to open further exploration of the role of values in different health decisions, political sectors besides health, and even other jurisdictions.

## Background

Values, defined as “*principles, or criteria, for selecting what are good (or better, or best) among objects, actions, ways of life, and social and political institutions and structures,* ”^1^ play an essential role in informing policy choices among options that could be used to achieve particular goals in health systems.^2-4^ In decision-making about health-system financing, social values, referred to sensibilities dealing with general social functioning^5^ such as efficiency, equity, quality, sustainability, and universality, are often embedded at all stages of the policy process but is not clear how they are incorporated.^6^ Moreover, understanding the role of those social values is challenging, given the wide range of values prioritized by different stakeholders and the many ways in which values can inform policy decisions.^7^

Latin America has provided a vibrant context for a study of the role of values given its many recent political transformations. In the last 40 years, many countries in the region have moved from dictatorships to democracies, democratic governments have been run by right-wing, centrist and left-wing parties and there has been unequal economic growth within and across countries. These factors have contributed to different values being prioritized in policy decision-making in each country and therefore used to shape the many efforts to reform health systems in the region.

Different authors have addressed the role of values in the health policy decision-making process in Latin America, principally focusing on values in health-system reforms.^8-10^ Those approaches were captured in our critical interpretive synthesis, which identified 116 values in empirical and non-empirical papers about the role of values in policy decision-making in Latin America. Through the critical interpretive synthesis we developed a theoretical framework, tested in this study, which characterized values in 4 ways: (1) goal-related values (ie, guiding principles of the health system); (2) technical values (those incorporated into the instruments adopted by policy-makers to ensure a sustainable and efficient health system); (3) governance values (those applied in the policy process to ensure a transparent and accountable process of decision-making); (4) situational values (a broad category of values that represent competing strategies to make decisions in the health systems). These categories of values come to be influential in government agenda setting by framing the problems in specific ways, by prioritizing some health issues in the government agenda, or by giving legitimacy to the process of agenda setting.^10^ In policy development, values are used as pragmatic instruments to inform policy development, influence what policy options are more likely to be chosen, and to improve the acceptability of the policy options that are selected.^11^ In policy implementation, values influence which policies are more likely of being prioritized for implementation, are used as indicators for evaluating the general performance of the health system and are used as indicators of good governance and as strategies against corruption.

Chile and Colombia are examples of 2 Latin American countries with a rich historical context for health-system financing decisions. Chile, in 1981, during the Pinochet’s dictatorship, and Colombia, in 1993, during a democratic government, reformed their health systems, introducing for-profit private health insurance companies.^11-13^ Since then, a two-tiered health system has existed in both countries.

In Chile, citizens can be affiliated either with a single national public insurer (the National Health Insurance Fund, Fondo Nacional de Salud or FONASA), or with private health insurance institutions called ISAPRES (Instituciones de Salud Previsional), which operate under the logic of premiums adjusted to individual risk.^14-16^ At the end of the Pinochet dictatorship in 1989, the period of transition to democracy was led by 4 consecutive governments belonging to a coalition of centre and centre-left parties. The first 2 transitional governments implemented minimal changes to the privately financed health system. As consequence of this “voluntary” and “market regulated” health system, in 2001, FONASA provided healthcare coverage for the majority (67%) of the Chilean population, while ISAPRES covered 19% of Chileans.^17^ As would be expected, those receiving coverage through ISAPRES are individuals from the top-two income quintiles, with a higher proportion of men and youth than the average Chilean population, which represent those with the lowest risk profiles in the population.^18^

In Colombia, the reform defined benefits packages to be provided under a national insurance system through an individual capitation scheme that includes a contributory regime for employees and a subsidized regime for unemployed and low-income families. The benefits of the subsidized basket of health services were approximately half the benefits in comparison to the contributory regime’s basket, which created an important source of inequity in the design of the system itself.^13,19^ An important contextual factor in Colombia is the introduction, in the Constitution of 1991, of the tutela, which is an informal and expedited injunction that allows individual claims for judicial protection when fundamental human rights are threatened by the state or by a third party.^13^ The introduction of this provision opened the door to a significant increase in litigation in relation to rights enshrined in the Constitution, including those related to the right to health,^20^ and strengthened the role of the judicial system in issues related to health policy, which provided the Constitutional Court a great deal of legitimacy in shaping health policy in the country.

In this research, we wanted to know: (1) what declared and undeclared values are important in the decision-making processes that crafted 4 particular policies? (2) how do policy-makers use values in these processes? and (3) why are some values incorporated?

## Methods

This study employed a qualitative multiple-case embedded design^21^ with 2 cases and 2 embedded units within each case. The choice of a multiple-case study design was driven by the comparative nature of our research question and the ability of this design to produce robust results.^21^ An embedded design was preferred given the nested nature of the context in which 2 different health-system financing decisions were made in each of the country cases.

### Case Selection

Within each country, 2 policy decisions were chosen as embedded units. Both decisions in each country needed to represent a significant shift in health-system financing policy as part of the health-system reforms. In each country, we selected one broad-reaching structural political decision (affecting financial arrangements as well as delivery and governance arrangements), and another with a narrower focus on resource allocation within the context of the broader structural reforms. Both of the structural decisions were implemented within the last 10 years, while the narrower decisions were made (since 2015). For Chile, we selected the development and implementation of the Universal Plan of Explicit Entitlements (AUGE or garantías explícitas de salud [GES]), which is a universal care plan designed to make medical coverage available to all Chilean citizens suffering from one of a specified, but growing list of diseases (80 at 2018). For the second decision, we selected the approval of a law mandating universal coverage of technologies for high-cost diseases (known as the Ricarte Soto Law), which provides financial protection for technologies associated with specific high-cost diseases to all citizens regardless of sector affiliation (public or private) or socioeconomic status.

For Colombia, we selected the declaration of health as a fundamental right, which implies a change in the notion of health as a public service (first with the rule T-760 and later with the Statutory Law). For the second decision, we selected the mechanism established by the Health Ministry to explicitly exclude technologies that cannot be publicly funded.

### Selection of Participants and Sampling

The selection of participants ensured a balance of perspectives of people involved in the policy decision-making process for each of the decisions selected. We used three criteria to define the sample frame. First, the involvement in the policy decision-making process, defined as being directly involved in one or more of the decisions included in our case study and/or in the policy analysis and development process that contributed to the eventual decision. Second, the type of government in Chile and Colombia. Both countries have a presidential bi-cameral regime with a separation of powers under which the state is divided into branches (the legislative, executive and judicial), each with separate and independent powers and areas of responsibility. Third, a balance of perspectives of stakeholders and policy-makers from the national and local level, and in favour of and opposition to the decisions. Based on this, we created a set of categories of types of roles or positions that could have been involved in the decisions. These included policy-makers (ie, from the health ministry, the health technologies assessment institutions or commissions, public health authorities and local governments; congressmen; and judges in the case of Colombia) and stakeholders (ie, academic authorities, medical and other health professional associations, hospitals and insurance managers, and advocacy coalitions comprising the aforementioned groups). Using this categorization scheme, we developed a sample frame of participants from individuals identified through our previous knowledge of the decisions by searching government and organizational directories for those who might have been involved in the decisions, and by asking interview participants to identify others who were involved.^22^

The sample frame was used to select a purposive sample of participants to invite for a qualitative interview. The purposive sampling sought to achieve: (1) maximum variation to ensure a range of key informants was sampled based on their position (ie, a mix of policy-makers and stakeholders); and (2) the engagement of individuals who could offer rich insights (eg, those who played multiple roles). We planned to interview between 12-24 participants in each country to ensure a breadth of perspectives across each of the policy-maker and stakeholder groups (roughly 6-12 interviewees from each group in each country) and to reach thematic saturation (ie, when no new themes emerge from the last 2-3 interviews).

### Field Procedures

We used an in-depth semi-structured interview approach to explore participants’ views and experiences about the three objectives of our study.^23^ Participants were contacted by email with follow-ups by email and then by phone call when necessary and invited to participate in a semi-structured interview of an approximate duration of 60-90 minutes. Those who agreed to participate were interviewed in-person, or by video-call when an in-person interview was not possible.

The principal investigator (MV) performed all the interviews, using a semi-structured interview guide. We provided several examples of values commonly related to health policy decision-making, including those explicitly stated in the laws that organize and regulate the health systems in Chile and Colombia. The interview guide included questions related to the general process of decision-making about health-system financing and about each of the decisions included. In each of these three areas, we included prompts related to each of the three questions. We iteratively revised the interview guide as needed to allow for exploration of emerging themes and to explore assumptions or statements made by other participants.

Each participant was asked to review and sign an informed consent form and was asked permission to record the interview. The audio recordings and transcripts were stored on a secure password-protected laptop. Interviews were conducted, transcribed and analysed in Spanish, and translated to English during the process of drafting the analysis of the results.

### Data Analysis

A thematic analysis was conducted using a constant comparative approach for each case study. Information was coded using three frameworks: (1) Kingdon’s government agenda-setting framework, which uses three ‘streams’ of factors (problems, policies, and politics) to explain why some issues garner government attention and why some issues are elevated to the point of being up for active decision^24^; (2) the 3I+E framework, which focuses on the role of institutions, interests, ideas, and external factors in shaping policy choices^25^; and (3) the values framework discussed previously.

First, data was examined through an open coding process by the principal investigator (MV), the codes were reviewed by grouping themes that are similar theoretically or connected in meaning, using the three frameworks outlined above. We created a list of codes that consisted of a catalogue of themes, issues, accounts of values, and opinions that relate to the process of policy decision-making across the cases. Based on these codes, the computer program ATLAS.Ti was used to generate a series of categories arranged in a treelike structure connecting text segments grouped into separate categories of codes or ‘‘nodes’’ to further the process of axial or pattern coding to examine the association between different a priori and emergent categories. The a priori categories were determined using Kingdon, the 3I+E framework, and our societal values framework, as the guide for the initial analysis. Then based on the initial coding, additional themes were derived and used to further code the data. MGW and GP reviewed the coding process and reassigned categories when necessary. A brief summary was sent to one participant in each country as strategy of member checking of the data analysis process.

Finally, we developed policy analyses in each country by: (1) identifying what values have informed the policies selected, and to understand how and why those values were used in that specific context; (2) describing stakeholder and policy-maker perspectives and insights about specific values identified in the interviews; and (3) comparing how these perspectives differ.

## Results

Forty stakeholders and policy-makers were invited to participate in the study (20 in each country), and a total of 28 key informant interviews were completed (9 in Chile and 19 in Colombia) (see characteristics of participants in Table 1). From the group of 12 individuals who were invited but did not participate, 5 declined, 5 did not reply, and 2 could not participate due to scheduling conflicts despite repeated attempts to find a time that worked for them. The interviews ranged from 33-108 minutes in length, with an average duration of 55 minutes (54 minutes in Colombia, 57 minutes in Chile).

**Table 1:** Summary of Participants by Role and Country

**Type of Participant**	**Chile**	**Colombia**
All participants	9	19
Policy-maker	3	7
Executive branch	3	4
Legislative branch	0	2
Judicial branch	0	1
Stakeholder	6	12
Manager	1	3
Health professional	6	11
Member of a coalition or stakeholder group	2	7
Researcher	6	6

Policy-maker and stakeholder are exclusive categories. Sub-categories of stakeholders are not exclusive.

In this section we provide a descriptive narrative of each of the decisions selected, identify what values were important in the decision-making processes that crafted those policies (ie, effectiveness, free-choice, human dignity, individualism, social participation, solidarity, stewardship, and sustainability); describe how those values were used (eg, framing problems in meaningful ways, guiding the policy development process, using them as pragmatic instruments to make decisions, and using them to legitimize decisions); and explain why these particular values were incorporated in the 4 decisions addressed.

### Main Findings in Chile

#### First Embedded Decision: AUGE/GES Plan

In the 1990s, politicians and stakeholders continuously criticized the performance of the Chilean health system given the feedback about how the system was failing to provide timely access to needed services. Values played a significant role in framing the problems of the Chilean health system and setting them on the governmental agenda. Feedback from the operation of current programs showed a lack of affordability for needed care due to limited to no coverage for certain diseases and health conditions and higher premiums for some populations (eg, older adults and women of child bearing age) in the privately financed system. In addition, poor quality of and lack of timely access to care in the publicly financed system was identified as a significant and persistent issue. Following the election of a new president (Ricardo Lago), the issue gained further prominence with his proposal of a health reform that would make the system more accessible, which appeared technically feasible and enjoyed public support in the aftermath of a recent election.

Several factors emerged from our analysis as being critical to supporting and also potentially limiting the likelihood of developing and implementing the AUGE/GES plan. The main factors supporting its development included: (1) the commitment to the proposal by the newly elected president Lagos, who were mostly unconstrained in their executive role; (2) the lobbying of patient groups who requested the inclusion of specific diseases to the plan (which helped to shape the focus of the plan on covering specific diseases and treatments for them); (3) the use of the right to health rhetoric in the discussion of the reforms which improved the acceptability of the proposal; and (4) the alignment of the plan with the proposal of basic universalism of the World Bank (as an external factor). In contrast, three factors appeared to have played against the development of the AUGE/GES plan: (1) the need to alter strong policy legacies of the health system established by Pinochet (which meant altering existing resources and incentives to groups in the system); (2) the opposition of private insurance companies to the solidarity fund proposed by the government (given that they would lose market share and revenue); and (3) the prevailing value of Chileans that access to private insurance as an indicator of improvement in their economic status and their social mobility (meaning that an enhanced public role could be seen as limiting their achievement of social mobility).

According to the interviewees, overcoming these competing factors to develop and implement the AUGE/GES Plan was achieved by careful balancing of the discordant values of free choice and individualism and the need for solidarity in the financing arrangements for the health system. Three mechanisms for how these values influence the decision-making were identified, which include framing problems in meaningful ways, guiding the policy development process, and as a way to ensure social mobility among citizens (see Table 2 for a description of how values were used). Regarding framing the problems, a contradiction between these sets of values appeared when considering that payroll taxes finance both the publicly and privately financed health systems. However, the publicly financed system is available and accessible for all Chileans, but in the privately financed system, employees use their payroll contribution to pay for individual private insurance (for the affiliate and his/her family), and these resources are not shared with the publicly financed system or with those who are sicker or have lower incomes. In guiding the policy development, solidarity was understood by some interviewees as the social duty of private-plan participants to share their contributions with all the Chilean population through a common fund. In contrast, interviewees understood the value of individualism as the desire of some Chileans to belong to an elite group with access to services that differentiate them from the general population. In this scenario, insurance companies pressed to maintain the status quo, because they could continue to benefit from the profits of this scheme in which high-income, low-risk individuals pay high premiums only to cover their own healthcare needs. Some of the interviewees stated that President Lagos’ proposal included a compensation solidarity fund as an attempt to reconcile these values, but this proposal lacked the political support of parties on the right of the political spectrum which, at least in part, represented the interests of private insurers. As a result, several actors consider that the reform ultimately took fell short in terms of seeking solidarity through the system.

**Table 2:** How Values Were Used in Each Embedded Decision in Chile

**Values Identified**	**Explanation of How the Values Were Used**
**Decision 1 - Regime of Explicit Health Guarantees (AUGE/GES Plan)**	
Goals-related values • Accessibility • Quality • Solidarity • Timely access	**To frame the problems of the health systems in meaningful ways for stakeholders and citizens** For example, solidarity was framed in this way: • Payroll taxes finance both public and private health systems in Chile. However, the public system is available and accessible for all Chileans, but in the private system, employees use their payroll contribution to pay for individual private insurance (for the affiliate and his/her family), and these resources are not shared with the public system, neither with those who are sicker or have lower incomes. ♦ *"But of course, if this guy is here [the private system], but tomorrow, the basic conditions for which he was there fail, and he came down, there was a network down there, as in the circus, then he fell off the trapeze, and fell on this basic network, which is the public system, which is your last-term insurance”* (Stakeholder 11).
	**To guide policy development from the perspective of an enforceable right to health** • The right to Health underpinned the specific goals-related values that guided the development of the AUGE/GES plan. Those goals were: (1) an enforceable right to health; (2) the definition of treatment protocols and specific interventions necessary for treating the medical condition [quality]; (3) the adoption of maximum waiting times for each condition [timely access]; (4) the adoption of limits on out-of-pocket spending according to the family’s income [accessibility]; and (5) the creation of a solidary fund to finance the public system [solidarity]. ♦ *" So, we recognize, first of all, that GES is a mechanism to make explicit the right to health, and therefore, it also makes explicit what is not guaranteed. So what was taken as philosophy was to say: you know what? we are going to make the right to health explicit, that is, we are not going to say that people have the right to health in generic terms and then we see what happens, but we are going to say what people are entitled to, and what is legally required, this means, legally established and enforceable"* (Stakeholder 11).
Technical values • Effectiveness • Evidence-based • Financial protection • Rationality • Relevance/importance to public health goals • Rationing	**Pragmatic instruments to develop the policy and decide which diseases and conditions should be included** • All interviewees agreed that the decision on which diseases and services to include in the AUGE/GES plan was informed by technical values, mainly considering effectiveness and relevance/importance to public health goals. **Pragmatic instruments to define the baskets of services that are funded for each condition included** • The definition of the benefits basket for each disease that can be funded, either with public resources in FONASA or with premiums in the ISAPRES, depended on a combination of clinical effectiveness and budgetary limitations to cover clinically effective services. **To legitimize decisions that were meant to deflect attention from budget constraints** • In defining what was included and excluded from the baskets of services, the value of rationing was weighted more than the value of rationality and, as a result, stakeholders perceived that the creation of some baskets of services lacked common sense and hid budget constraints (Stakeholders 11 & 12 and Policy-maker 14). ♦ *"We said OK cataract, let's operate on cataracts, but we said let's operate only on one eye. But how? if there is an X percent who has bilateral cataracts, why are we going to operate on one eye and not the other? ... At that time the story was like this, it is better to have a one-eyed man than a blind man, there's no money for everything, so it is better to have a one-eyed man than a blind man, we are going to operate on one eye, and is very clear"* (Stakeholder 12).
Governance values • Accountability • Social participation • Transparency	**To legitimize the policy development process** • Participation of patients and citizens in the process of deciding on the conditions to be included in the AUGE plan improved the social perception about the capacity of the government to respond to social needs. • However, participation of patients and citizens also appeared to reduce the credibility and transparency of the process as a result of the seemingly disproportionate influence of some groups such as parents of children with cystic fibrosis. ♦ *"And that is what happens with the Holocaust phenomenon, when you see the film, and you focus on a case where a child is murdered; the pain, we all fall tears. But you know that millions died, and when you know that millions died, it's like the pain diminishes. It is a psychological phenomenon. But you cannot ask that to a normative body, a normative body is supposed to feel 10 times the pain when there are 10 cases, or 100 times or a thousand times, that is the difference between the normative and the emotional descriptive. So, what is happening here is that there is a value in positioning this index case. Then, you put a child with cystic fibrosis in the media, and it appears, it is in the minds of all individuals in the society, and then cystic fibrosis has to be, and you generate and occupy that psychological mechanism, and that psychological mechanism enters into the agenda and has an effect on society. Moreover, that happens because there is not a structured normative body filtering that, that allows putting that in the balance with other things, where there is probably more pain, in sum. What's the name of that social value? I do not know how it's called"* (Stakeholder 12).
Situational values • Free-choice • Individuality	**As a way to ensure social mobility among citizens** • Several Chileans consider the health insurance as a market (private) good that can only be accessed through an individual’s purchasing power. • Several interviewees stated that Chileans give importance to the free-choice of joining the private sector, because an improvement in their economic status that makes them eligible to belong to the private health system is an indicator of social mobility. ♦ *“Then, you have a society like this. Suddenly one of the trapezes falls, and suddenly we see some of those who are here [down] who improve the conditions; guess what they do? He quickly moves here [for the private one]. Why? Because he escapes the vices and complications of the public system, which are typical, the waiting list, the deal, and that ... somehow, let's say, his change of social status has attached the sticker: I´m in ISAPRE, this is part of his social repositioning"* (Stakeholder 11).
**Decision 2 - Fund for Health Coverage of High-Cost Diseases (Ricarte Soto Law)**	
Goals-related values • Equity	**To frame the problems in meaningful ways for stakeholders and citizens** • The journalist Ricarte Soto framed the problem of the lack of coverage of treatments for high-cost diseases as an equity problem and highlighted that patients in the private system had timely access to those technologies but patients in the public system faced several barriers.
Technical values • Effectiveness • Financial protection • Safety	**Pragmatic instruments to develop the policy, decide which diseases and conditions should be included and to define the technologies and services covered** • The criteria to decide which diseases were included in the Ricarte Soto Fund was the financial impact that treatments caused to families. To decide which technologies to cover with the fund, the criteria was the effectiveness and safety of the interventions. Given that the treatments are high-cost, values such as cost-effectiveness or disease burden were not prioritized in making this decision. ♦ *"I strongly would say that financial protection. The fact that nobody can die for not having a way to pay for her high-cost treatment, regardless of whether it was rare or not rare diseases. That was the strongest concept, and that is why the central proposal was to create a drug fund. It translated into what do we do as a country to protect these people from having their lives go to ruin by having to pay for these expensive medicines? Moreover, hand in hand, those who are most affected, that are the poorest, the middle class, and therefore the proposal had a content of equity, then it is like a mixture between both, and from that citizen´s feeling was that the public policy born in this government"* (Policy-maker 17).
Governance values: • Accountability • Citizen engagement • Social participation • Transparency	**To prioritize which conditions and technologies to include** • The participation of different patients’ groups in the process of prioritization implied discussions among those participants and the policy-makers to decide which conditions and technologies would be funded with the Ricarte Soto Law. **To improve the acceptability of the policy** • Participation of patients and citizens in defining the conditions included in the Ricarte Soto Fund improved the social perception about the capacity of the government of giving answers to the social needs.

Abbreviations: FONASA, The National Health Insurance Fund, Fondo Nacional de Salud; ISAPRES, Instituciones de Salud Previsional; GES, garantías explícitas de salud; AUGE, Universal Plan of Explicit Entitlements.

All interviewees agreed that the decision on which diseases and services to include in the AUGE/GES plan was ultimately guided by a focus on technical values, particularly relevance/importance to achieving public health goals for the first, and effectiveness, financial sustainability, and budget availability, for the decision about what to include in the covered basket of services. Those values where used through 2 mechanisms, as pragmatic instruments to make decisions and as strategy to legitimize decisions were meant to deflect attention from budget constraints.

Interviewees also highlighted that the inclusion of cystic fibrosis as one of the first 25 diseases included in the AUGE plan, was influenced by the engagement of patients and citizens, principally, by the mobilization of the interests of parents of children with the disease. Interviewees indicated that the inclusion of cystic fibrosis arose a contradiction in the policy-development process between technical values prioritized by policy-makers and social values advocated for by civil society. For some policy-makers, decisions that prioritize social values advocated by civil society, led people to view the policy process as lacking accountability, credibility, and transparency, given that interest groups influence the decision. In contrast, stakeholders and some policy-makers considered that decisions only informed by technical values lack accountability, credibility, and transparency.

#### Second Embedded Decision: The Ricarte Soto Law

The Ricarte Soto Law emerged as a response to a focusing event. When the journalist Ricarte Soto was diagnosed with lung cancer he became aware of the economic barriers that patients like him face when trying to access needed treatments, particularly high-cost treatments not included in the AUGE/GES Plan. This awareness led the journalist to promote a social movement advocating for a policy that guarantees access to treatments for high-cost diseases, which subsequently garnered political support in the form of a programmatic proposal of the presidential candidate, Michell Bachelet, who promised a fund to cover drugs associated with complex and high-cost diseases.^26^

Four factors supported the development of the Ricarte Soto Law: (1) the governing party supported the initiative (ie, it was programmatic proposal of the newly elected president); (2) the social mobilization and lobbying efforts of patient groups made highly visible requests for the coverage of specific technologies; (3) intense media coverage fostered further support among the Chilean population, which was a key factor in changing the national mood to support the initiative; and (4) the need for the newly elected president Bachelet to develop a policy that improved her public image and helped her recover support among citizens (given than in other political areas she was losing favourable public opinion). Given that the Ricarte Soto proposal did not affect the profits of insurance companies nor the public budget of the government, the proposal had little opposition.

According to the interviewees, the key values that underpinned the proposed policy were equity and, to a lesser extent, social participation. Those values were used to framing problems in meaningful ways, guiding the policy development process, and as strategy to legitimize decisions. Equity was identified in relation to financial protection (ie, that need and not ability to pay should determine coverage for high-cost diseases), and also in relation to access to services irrespective of type of disease (ie, that patients with rare diseases should be able to access treatments they need in the same way that patients with more prevalent medical conditions). Social participation was identified as the other significant value in the context of the importance of being responsive to social requests for policy action to address an issue and its prioritization ultimately rewarded the government with citizen support and favourable public opinion.

Overall, most of the interviewees indicated that the focus of health policy decision-making should be on optimizing benefit to society as opposed to making decisions to solve individual cases. Accordingly, if the focus is to make decisions that benefit society, interviewees argued that technical values such as disease burden (ie, disease prevalence and severity of the conditions), and the effectiveness and cost-effectiveness of interventions to address them should be used by policy-makers and stakeholders. However, policy-makers and stakeholders stated that clear technical criteria to balance differing interests in the system do not exist, which makes robust citizen engagement important to help ensure transparency and accountability in the face of competing technical and societal interests.

#### Main Findings in Colombia

##### First Embedded Decision: Declaration of Health as a Fundamental Right

Between 1999 and 2008 the number of tutelas filed regarding health claims had increased by 300%.^27^ Some of these tutelas included claims for healthcare services “excluded” from the benefits plans of each regime. However, most of the claims were to enforce coverage for technologies and services already included in benefits plans that health insurance companies unlawfully denied.^19^ Most of these tutelas were granted to the plaintiffs, which made judges the last recourse to decide which health benefits citizens are entitled. This situation and the lack of compliance with the process established in the transitional rules of the health system in 1993 for updating and equalizing the contributory and subsidized benefit plans by the year 2002, led to the active role of the Constitutional Court, who seeking to reconfigure the social values with which health policy decisions are made, mandated the right to health as the focus of decision-making about health policy in Colombia. As a result, the judicial sector was the central driving force behind the focus on upholding the right to health being prioritized on the decision agenda of the Colombian government.

In 2008, the Constitutional Court passed the Rule T-760, which ordered a series of structural changes to the health system. For instance, the Court ordered the government to take the necessary steps to unify the 2 regimes of health coverage, and to update the benefits included in the new unified plan. While the Government timidly began a process of elaborating plans and policies to comply with the orders of the Constitutional Court, the private insurance companies continued to deny services included in the benefits plans, and as consequence tutelas continued to increase between 2010-2014 to approximately 107.000 each year.^28^ Preceded by 2 failed policies developed to solve these problems in 2010 and 2011 (the Social Emergency in Health and the Law 1122), the government of Juan Manuel Santos presented a Statutory Law bill to the Congress in 2013, which passed after changes introduced by the Constitutional Court. The Statutory Law declared health as a fundamental right and de-incentivized litigation on costly healthcare services by forbidding the use of public funds to reimburse cosmetic and experimental medical treatments demanded by patients.^29^

The most important values in this embedded decision were human dignity, financial sustainability, and stewardship. Four mechanisms for how values influence the decision-making were identified, which include framing problems in meaningful ways, guiding the policy development process, to gain legitimacy in the policy-making process in the absence of meaningful citizen participation, and to shape policies in a way that is aligned with the ideas of an influential interest group (see Table 3 for a description of how values were used).

**Table 3:** How Values Were Used in Each Embedded Decision in Colombia

**Values Identified**	**Explanation of How the Values Were Used**
**Decision 1 - Declaration of Health as a Fundamental Right**	
Goals-related values • Equality • Quality • Right to health • Universality	**To frame the problems of the health system in meaningful ways for stakeholders and citizens** For example, right to health was framed in this way: • The Constitutional Court inferred the right to health from the right to life (doctrine of connection). The meaning was that although right to health was not declared as a fundamental right in the Constitution, it could become fundamental and enforceable by its connection to the right to life. ♦ *"But when the Court realized that there were many concrete cases in which the principle that the court believed was the first or of value, was disregarded, then it said, all this is bad, it is no longer this concrete case, it is all the system, all is wrong, is not working"* (Stakeholder 16). **Value-based framing of problems shape viable solutions to achieve desired goals** • The problem was primarily framed in relation to the lack of stability and financial sustainability of the Colombian health system, as well as an overburdened judicial sector from the significant increase in tutelas, which ultimately exposed the more general flaw in achieving the protected value of the right to health in Colombia. • Within this framing, 2 sets of policy options emerged which focused on reducing the number of tutelas by: 1) Controlling what is requested by doctors and monitoring, auditing and regulating the payments of reimbursements due to tutelas (informed by values ​​of sustainability, protection of the state resources, and the enforcement of regulation); and 2) Protecting the right to health, updating the baskets of benefits, and ensuring appropriate healthcare delivery to citizens (based on values ​​such as accessibility, continuity of healthcare, comprehensiveness, equity and ‘human dignity’ as it relates to the judicial branch).
Technical values • Efficiency • Evidence-based • Sustainability	**To achieve the goals of the health system with efficiency and sustainability** • The Constitutional Court considered technical values ​​as necessary instruments to achieve the right to health and assurance of human dignity. ♦ *"I think we have to tune the two, what is important is that sustainability is not an end in itself, it is an aim precisely to achieve the protection of rights, and it has to be taken very seriously and not be regarded as a general argument. As we know resources are missing, as we know there are these limitations, then proceed; it has to be specific"* (Policy-maker 22).
Governance values • Enforcement of regulation • Social participation • Stewardship • Transparency	**To frame the problems as lack of stewardship, enforcement of regulation and corruption** • Several stakeholders pointed to the lack of conflict resolution mechanisms that could address the struggles in the health system by preventing the escalation of litigation and enforcing regulations designed to implement the legislation for the health system. **To gain legitimacy in the policy-making process in the absence of meaningful citizen participation** • Stakeholders highlighted that the large number of guiding values ​​of the Colombian health system (currently 27) meant that decision-makers can easily define policies in relation to one or more of these goals thereby gaining legitimacy without meaningful engagement of citizens. ♦ *"You see that Chile has some very specific ones [values], very well defined, but in Colombia, you do not see that, you see the desire to include and include principles ... and that, at the moment of the truth, is not practical for Colombia. When you start looking, you can use anything, because the principle is there, whatever you want, you can do it because the principle is there, there are so many, that anything can be useful, whatever you want will serve anybody. I think it's the big mistake that Colombia has. It is easy, you first do and then look what value fits, it's the same when you set 80 goals, you can do anything that you want because it will point you to any goal. What you set can work accordingly for any goal"* (Stakeholder 28).
Situational values • Gradualism • Human dignity	**To shape policy development in a way that is aligned with the ideas of an influential interest group** • The Constitutional Court has given priority to the value of gradualism and human dignity. Gradualism has been defined as the progressiveness in the development and expansion of the protection of the right to health. Human dignity has been understood as the right to healthcare that allows an individual the physical and psychological integrity and empowerment. Those values have shaped health policy in a way that requires that it must always result in more services and technologies funded with public resources, and more people covered with those benefits. ♦ *"It was reasonable that the benefits were full for those contributing for themselves and others, and that, after a time, when the sustainability of the system allowed, then, the benefits would be equal for all. That is reasonable, it is reasonable to restrict equality and equity for a time for reasons of sustainability, in order to reach equality, there is when this makes sense. Moreover, that is the way to harmonize them, but not as an irresponsible way of thinking that I can protect rights without any kind of economic reality and so on. Or the opposite, that it is an end in itself, and forgetting that this sustainability is being pursued to reach all people with equality and dignity"* (Policy-maker 22).
**Decision 2 - Mechanism of Exclusion of Technologies to be Funded With Public Resources**	
Goals-related values • Equity • Right to health	**To frame the problem as a meaningful social concept for stakeholders and citizens** • Expensive technologies funded for low-prevalence diseases and only for citizens who can navigate in the judicial system (mainly middle and upper-middle classes) result in communicable and prevalent diseases in impoverished regions that are not supported by the system, which resulted in the recognition that the collective right to health was not ensured.
Technical values • Cost-containment • Effectiveness • Evidence-based • Safety • Sustainability	**Influence the government agenda because the economic stability of the health system was at risk** • According to policy-makers, the Statutory Law practically ordered public funding for all technologies and services, risking the stability of the health system. This resulted in the need for a mechanism of exclusions in order to be able to also ensure the value of sustainability. **As pragmatic instruments to define the technologies and services covered** • A key example of how values were used as pragmatic instruments was in the discussion about the coverage of reconstructive surgery for women with breast cancer as the decision of the Court was that the nature of cosmetic or reconstructive surgery would be decided on scientific criteria and not supported by administrative or financial considerations of the insurance companies or the patient’s opinion. The court highlighted that cosmetic surgery is expressly excluded, while reconstructive or functional surgeries are understood to be included and under the responsibility of the insurance companies.
Governance values • Citizen engagement • Social participation • Transparency	**To improve the acceptability of the policy** • The government considered successful national and international experiences with social participation and citizen engagement to improve the acceptability of the policy. However, patients’ organizations, health professionals, and social organizations fear that participating implies endorsement of policies with which they disagree.
Situational values • Human dignity	**To shape policy development in a way that is aligned with the ideas of an influential interest group** Patients, health professionals, and social organizations criticize the development of the mechanism of exclusions given they consider the value of human dignity was converted into rigid technical criteria. They advocate for the decisions to respect this value promoted by the court. ♦ *"How is this interpreted in reality? Everything cosmetic is excluded? the Court even puts a particular example, a child with prominent ears, because there was a tutela. The prominent ears do not cause any problem to the kid, the kid is healthy and can make a living, but is that other kids are bullying him, they are damaging their self-esteem, he will have emotional or psychological sequels. Is this case the same as the case of a lady who simply wants to have an augmentation mammoplasty to look better? Well, no. Those are 2 completely different cases"* (Stakeholder 2).

According to the interviewees, the increase in tutelas was framed in terms of a threat to the financial sustainability of the health system (given the expensive payments for reimbursements to the private insurance companies), and as a violation of human dignity (given that the legal rules for the provision of healthcare were not being upheld and judges had to intervene on individual cases). Some of the policy-makers interviewed (belonging to the executive branch) criticized the policy development promoted by the Constitutional Court, arguing that it only considered the value of human dignity and neglected the value of financial sustainability, they suggested that the Court supported the right to health in theory but did not define the limits of that right. This meant that any request for direct or indirect healthcare ended up being protected by the right to health under the principle of human dignity (eg, diapers, food, payment of salary to family caregivers, etc), which put the financial sustainability of the health system at risk. For other policy-makers (belonging to the judicial branch) and for the stakeholders, financial sustainability was relegated to a factor that helps to guarantee the right to health for all.

All interviewees emphasized the importance of placing explicit boundaries on the package of benefits to which Colombians are entitled given that the health system is unable to provide everything to everyone. Some participants pointed out that by means of tutelas and allegations, expensive technologies were being funded for low-prevalence diseases and only for citizens who could navigate the judicial system (mainly middle- and upper-middle classes). Therefore, communicable and prevalent diseases in impoverished regions were not being addressed, which was highlighted as an important violation of the collective right to health at the expense of assuring individual rights to health through those seeking benefits through tutelas.

While emphasizing the importance of establishing limits to the benefits basket, participants identified 5 factors that made it complicated to achieve: (1) distrust of stakeholders and policy-makers about the hidden interests of other stakeholders and policy-makers (eg, corruption, pursuing of additional profits); (2) difficulty in establishing social participation mechanisms (eg, with citizens, patients, interest groups) that ensure transparency and meaningful consideration of all the interests of interested parties; (3) perception of citizens, patients and doctors that giving up benefits (from the benefits basket) is unfair in view of that private insurers and private providers are not giving up part of their profits; (4) perception that scientific evidence or technical criteria used to define the benefits baskets are tied to the interests of the medical industrial complex or that they do not consider the values and preferences of the citizens; and (5) fear that health resources will be lost through corruption and not used to cover the real needs of the population.

This mistrust among health system actors is fueled by the low level of social participation in the development and implementation of policies regarding the financing of the health system and using guiding values as ‘shields’ for engaging in meaningful deliberation. The policy legacy of power concentrated in the executive branch of government through the president meant the government could avoid putting in place real spaces for discussion and consideration of policies that solve health system problems. Since the 1990s, social participation has been encouraged, but the government has proposed mechanisms and moments of participation that are more about communicating the decisions than participating in their development. This phenomenon has led stakeholders (and some members of Congress) to reason that a request from the executive to participate or deliberate implies a request for them to legitimize or endorse a particular course of action, and that any contradictory position is not taken into account. In addition, stakeholders also highlighted that the large number of guiding values in the Colombian health system (currently 27) are being used as a shield against having to engage in meaningful democratic development of policies. As a result, decision-makers define any policy and subsequently link one or several of these guiding values to the policy in an attempt to legitimize the lack of social participation and citizen engagement.

##### Second Embedded Decision: Mechanism of Exclusion of Technologies

The Statutory Law mandated the executive to develop and implement a technical-scientific mechanism to define those services excluded from the core of the right to health, which would mean that patients were entitled to all technologies and services except those that are specifically excluded from the benefits plan.^29^ Feedback from the operation of current programs highlighted to the Court that previous definitions of benefits packages lacked transparency and the use of evidence-based mechanisms for prioritizing what technologies should be publicly funded. To solve this problem, the Court asked for a public, collective, participatory, and transparent technical-scientific procedure, which would consider 5 guiding principles for excluding technologies and procedures from public funding. These included: (1) procedures considered cosmetic, (2) procedures or technologies considered experimental, (3) technology without scientific evidence of effectiveness, (4) technology without approval of health authorities, and (5) procedures to be provided abroad when available in Colombia.

The mechanism for exclusions was developed in 2017 through Resolution 330 of the Ministry of Health.^30^ The Ministry led the process through which it promoted that stakeholders submit technologies that should be excluded, and engaged them in deliberative meetings to decide which technologies should be prioritized for exclusion from publicly funded benefits.

In this decision, policy-makers and stakeholders identified one similar value prioritized during the agenda-setting process and policy development (ie, financial sustainability), but they differed in the other prioritized values. For policy-makers, the development of the mechanism for exclusions was informed by values such as effectiveness, financial feasibility, scientific evidence, universality, and values and preferences of citizens, while stakeholders prioritized cost containment and the profitability of different private actors as key values in the policy process. These discordant points of view about the values informing the policy process negatively affected the acceptance of the mechanism for exclusions by several stakeholders, who consider that it does not reflect the values promoted by the Constitutional Court (eg, gradualism and human dignity). These discordant points of view also led to the erosion of policy-makers’ confidence in social participation processes, and their view that citizens, patients, and doctors are not ready to be involved in the policy process, and that many Colombian citizens lack understanding of what a public good like healthcare means and therefore, they are not prone to protect the resources of the system.

For several stakeholders, the mechanism for exclusions proposed by the Ministry was inadequate. Specifically, interviewees argued that the Ministry converted the guiding principles ordered by the Court into rigid technical criteria, which they saw as being contrary to the spirit of the statutory law where each case should be defined according to the value of human dignity. In contrast, policy-makers viewed the issue in relation to the significant financial instability of the health system where virtually everything could be required to be publicly funded with resources from the health system.

Overall, this distrust in the process of policy development created a barrier to carrying out the orders of the Court. For example, stakeholders rejected many policies developed by the Ministry of Health because they perceived that international organizations (eg, WB, IMF, IDB) provided the ‘recipe’ for health policies and that the Ministry of Health and other governmental institutions simply adapted the ‘recipe’ to the context. In addition, stakeholders opposed the policies developed by the Ministry as they viewed policy-makers as traditionally only having considered options that seek to reinforce the existing private health insurance model without considering whether those policy options can potentially solve the problems on the agenda.

## Discussion

The values more frequently and strongly explained by the interviewees in this study were individualism, free-choice, and solidarity in Chile; human dignity, financial sustainability, and stewardship in Colombia; and effectiveness and social participation in both countries. We identified individualism in Chile as the only undeclared value (value not explicitly mentioned in policy documents or media), as well as undeclared interpretations of values explicitly named in policy documents (ie, the perception that social participation negatively affects the consideration of cost-effectiveness in decisions).

In general, we identified 4 mechanisms for how values influence the decision-making, which include framing problems in meaningful ways, guiding the policy development process, using them as pragmatic instruments to make decisions, and using them to legitimize decisions. In Chile, we identified that values were also used as a way to ensure social mobility among citizens, and specifically to legitimize decisions that were meant to deflect attention from budget constraints (see Table 2). In Colombia, we identified that values were also used to gain legitimacy in the policy-making process in the absence of meaningful citizen participation, and to shape policies in a way that is aligned with the ideas of an influential interest group (see Table 3).

Comparing both countries and explaining why some values are incorporated in the policy-making process, we found that in the case of Chile, from policy-makers and stakeholders emerged that citizens are very attached to the values of free choice and individualism, which meant that they prioritized being able to join private plans. In contrast, while Colombia shares similar contextual and political characteristics with Chile, individualism was not mentioned in any of the interviews in Colombia. This difference may be explained by the influence of policy legacies, given that Chileans can voluntarily join the publicly financed or privately financed health system, while in Colombia affiliation with the publicly financed and privately managed health system is compulsory for all. While individualism appeared to play no role in the 2 Colombian decisions studied, this value could still be relevant in Colombia if another topic (eg, prepaid medicine plans) were discussed, given that the people who commonly can afford these plans are recognized as belonging to the high-income population.

In the case of Colombia, the value of human dignity was incorporated in the policy decision-making process because of the judicial activism of the Constitutional Court. According to the interviewees, power in Colombia is concentrated in the executive branch of government (specifically, in the office of the president), which has prioritized financial sustainability over other values. The Court acted as a constitutional check on the decisions of the executive and legislative branches when it considered that the lack of the value of human dignity in the policy decision-making process was not living up to the branches’ constitutional requirements, and therefore must be included. This finding is similar to what Landau et al suggests as an explanation for the strong influence of the Constitutional Court in Colombia. According to Landau, the Court has responded to the instability of the political parties, poor social participation, and the weakness of the Congress in proposing policies and checking presidential power.^31^ Moreover, it is said that the strong role of the judiciary branch is a signal of new or weak democracies, and in all cases, this kind of legislative substitution is inappropriate for the policy decision-making.^31,32^

The smaller set of prioritized and often competing values identified in the 4 decisions, relates to how policy-makers and stakeholders consider them in decision-making and simplify the complex interplay influencing a particular decision to a few elements (values) that represent the extremes of the spectrum of points of views and policy alternatives to solve a problem; for example, the current law that organizes the health system of Colombia has 27 guiding values, which are impossible to address in each policy decision; however, dichotomizing values in tension facilitates stakeholders and policy-makers to identify and prioritize essential values in the policy process.

In a recent study about the values of public officials (not only in the health system), authors found that tensions between values and value trade‐offs are frequently founded in the public sector, principally when the decision-making imply allocation and redistribution of resources.^33^ In the analysis about the trade-off between efficiency and equity, authors suggest that public officials closer to the recipients of policies, are more pro-equity oriented, while public officials that have been exposed to private sector management, are more pro-efficiency oriented.^33^ Our results are consistent with those of Fernández‐Gutiérrez and Van de Walle,^33^ but highlight a new actor as policy-maker, the judiciary sector. In our study, interviewees from the executive branch were more oriented to prioritize financial sustainability and efficiency, while interviewees from the judiciary branch were more oriented to human dignity and equity.

Our study results should be interpreted carefully given 2 important limitations. First, the number and composition of the sampling of participants in Colombia had twice the number of interviewers than in Chile, which was partially driven by us inviting more participants to engage a broader array of people with different ideological positions and professional backgrounds given the significant polarization of the perceptions about the health system in Colombia, and the role of the Judiciary branch. In Chile, the agreement between different views of the health system and the values that inform the decisions were greater and, as a result, we reached saturation with fewer participants. Second, the imbalance in participants from each country, given that in Colombia we interviewed policy-makers belonging to the judiciary, legislative, and executive branches, while in Chile we only interviewed policy-makers from the executive. The principal reason for this is that the judiciary branch is not involved in the health policy decision-making in Chile and that during the fieldwork for this study Chile was in an electoral period and, as a result, it was not possible to conduct an interview with a member of congress during that period of time. This limitation was minimized given that we reviewed the transcriptions of public debates about AUGE/GES plan in the Chilean Congress, and we did not identify a divergence between the statements of members of Congress in those hearings and the insights of policy-makers participating in this case study.

## Conclusion

The study of values in the policy decision-making process in Latin America is an emerging field. Our effort to analyze health-system financing policies in Chile and Colombia using analytical frameworks related to government agenda setting, policy development and implementation and by considering the influence of societal values is a unique contribution to the body of knowledge in this field. As such, it is an opportunity to open further exploration of the role of values in different health decisions, political sectors besides health, and even other jurisdictions.

## Ethical issues

Three different research ethics committees approved this study: 1) the Hamilton Integrated Research Ethics Board (Canada) (protocol #2388); 2) the ethics committee of the University Adolfo Ibáñez in Santiago de Chile (protocol #2017); and 3) the ethics committee of the Faculty of Medicine of the University of Antioquia in Medellin (Colombia) (Protocol F-017-00). Each policy-maker and stakeholder interviewed provided consent using the information and forms approved by these ethics committees.

## Competing interests

Authors declare that they have no competing interests.

## Authors’ contributions

MV designed the study, made the data collection, performed the analysis, and drafted the manuscript. MGW participated in the study design, data analysis, and critically reviewed the manuscript. JA participated in the study design, data analysis, and critically reviewed the manuscript. JNL participated in the study design and critically reviewed the manuscript. GP participated in the study design, data analysis, and critically reviewed the manuscript.

## Authors’ affiliations

^1^McMaster Health Forum, McMaster University, Hamilton, ON, Canada. ^2^Department of Health Research Methods, Evidence and Impact, McMaster University, Hamilton, ON, Canada. ^3^Centre for Health Economics and Policy Analysis, McMaster University, Hamilton, ON, Canada. ^4^Health Policy Ph.D. Program, McMaster University, Hamilton, ON, Canada. ^5^Department of Paediatrics, Faculty of Medicine, University of Antioquia, Antioquia, Colombia. ^6^Department of Political Science, McMaster University, Hamilton, ON, Canada. ^7^Africa Centre for Evidence, University of Johannesburg, Johannesburg, South Africa. ^8^Business School, Universidad Adolfo Ibáñez, Santiago, Chile.

## Key messages

**Implications for policy makers**

- Understanding how social values are incorporated into health policy decision-making is important for policy analysis.
- The interplay between technical, social, and political values are important to designing policies that meet the needs of the population and responding to citizens’ preferences.
- Policy-makers simplify the complex interplay of values by prioritizing only those considered essential for the policy process.

**Implications for the public**

All policy decisions are value-laden; however, it is not clear how values play a role and why some values are prioritized. This study analyzes the values prioritized in 4 health system financing decisions regarding the materialization of the right to health, the designing of benefits plans, and the decision of publicly fund high cost technologies. This study supports the public understanding of how particular proposals or initiatives should align with the national mood to influence the decision-making.
